# Altmetric and Bibliographic Analysis of the Most Popular Articles on Vitreoretinal Surgery Between 2010 and 2020

**DOI:** 10.7759/cureus.36465

**Published:** 2023-03-21

**Authors:** Mevlüt Yılmaz, Osman Ahmet Polat

**Affiliations:** 1 Department of Ophthalmology, Ankara Etlik City Hospital Integrated Healthcare Campus, Ankara, TUR; 2 Department of Ophthalmology, Erciyes University Faculty of Medicine, Kayseri, TUR

**Keywords:** traditional metrics, ophthalmology, vitreous, retina, vitreoretinal surgery, altmetrics

## Abstract

Purpose

Altmetrics is a web-based metrics method that measures the online dissemination of an article and the interactions it receives. We aimed to perform a bibliometric and altmetrics analysis of the 100 most cited articles (T100) on vitreoretinal surgery (VRS) published between 2010 and 2020.

Methods

A detailed search including terms regarding VRS in the Web of Science database was conducted. Articles were examined for bibliometric data and altmetrics.

Results

T100 articles had citation numbers ranging from 55 to 368 and altmetrics score (AS) values ranging from 0 to 125, and there was no statistically significant correlation between them. AS was weakly correlated with publication year. A statistically weak negative correlation was also found between AS and percent of citable open access, number of years since publication (NYsP), and average citations per year (ACpY).

Conclusion

AS values of articles on VRS were found to be low. Apparently, this was because the issue was of a highly specific and technical nature. However, the existence of articles with a zero AS value despite high citation numbers suggests that journals and authors do not yet attach enough importance to altmetrics. Altmetric analysis is not a reliable indicator for evaluating the scientific value of an article, and it cannot be a substitute for traditional metrics but it can provide perspective on the social impact of articles.

## Introduction

The main purpose of academic publishing is to convey scientific knowledge to a broad audience. Today, the extensive use of social media and various online platforms facilitates faster dissemination, sharing, and even discussion of scientific outputs [1,2]. The impact of articles is evaluated with various metrics measurements. The most important factor that traditional metrics take into account is the number of citations that an article receives. However, for even a very high-quality article, it takes a certain amount of time to reach high citation numbers [3]. Meanwhile, the article may lose its actuality, or the attention of the relevant audience may divert away from the topic of the article [4]. As a consequence, there may be a decrease in the influence the article could create.

Today, in the era of the internet and social media, alternative metrics methods are needed for the evaluation of scientific articles in addition to traditional metrics. Altmetrics is considered a practical method for the estimation of the impact of research studies on the internet [5,6]. Altmetrics is a web-based metric system and has been developed as an adjunct to traditional metrics to show the impact of studies from different aspects [7]. Some academic and funding organizations now accept altmetrics as alternative forms of impact [8].

Altmetric analysis focuses on monitoring the impact of a publication in the online world rather than the academic world, and it allows us to measure how much a scientific study is mentioned on various online platforms such as social media, blogs, news, and podcasts, along with the number of online views and downloads [9]. A key advantage of altmetric analysis is that authors can track the online distribution of an article and get feedback on its online interactions thanks to the comprehensive reports it provides. Readers can also access post-publication comments collated on the article’s altmetric analysis page [8]. Altmetric measurements have been made through various online systems since 2010. One of the most widely used is the altmetric.com website, and the altmetrics score (AS) is generated by an automatic algorithm [9].

Vitreoretinal surgery (VRS) is one of the most sophisticated surgeries. The most common diseases requiring VRS are retinal detachments, vitreoretinal interface diseases, diabetes-related retinal complications, and ocular traumas [10]. Advanced surgical devices and instruments, high-resolution imaging systems, and a trained and experienced surgical team are required to perform VRS. In 1970, Machemer et al. performed a closed system pars plana vitrectomy for the first time. Since then, there have been many important developments in VRS technology, and it continues to evolve [11,12].

In this study, we aimed to analyze the 100 most cited articles (T100) on VRS in terms of altmetric and other bibliometric parameters.

## Materials and methods

The Web of Science (WoS) Core Collection database was selected in the WoS database. A search was made in the title and abstract sections using the keywords “vitrectomy OR vitreoretinal surgery OR pars plana vitrectomy OR retinal detachment surgery OR macula surgery OR retina surgery” in the basic search section. The time range was adjusted from 01.01.2010 to 31.12.2020. A total of 12,198 articles were obtained and ranked from highest to least according to the number of citations. The first 100 articles regarding VRS with the highest citation numbers were included in the study (access date: 29 May 2022; data were obtained from WoS and the InCites Journal Citation Report 2020). Detailed information on the scores obtained can be viewed at https://jcr.clarivate.com/jcr-jp/journal-profile?journal=OPHTHALMOLOGY&year=2020, with a domain name system (DNS) with WoS access.

**Figure 1:**
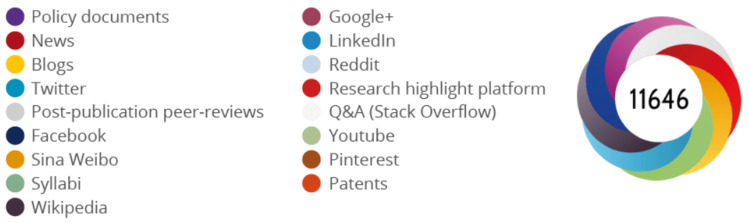
Altmetric score donut and its sources

The inclusion criteria of the study were as follows: VRS techniques, surgical instruments and materials, imaging methods, recent advances in VRS, prognostic factors, and results of VRS. Articles were evaluated in terms of authors, year of publication, total citation number, and average citations per year (ACpY). Bibliometric data of the journals that published the articles were recorded as follows: impact factor for 2020, five-year impact factor, h-index, immediacy index, Eigenfactor score, normalized Eigenfactor score, article influence score, journal citation indicator, ACpY WoS Core, percent of citable open access, ACpY all databases, and AS. All bibliometric data other than AS and h-index were obtained from the WoS Core database. H-index was obtained from the SCImago Journal list. AS of the articles were obtained with a free web browser bookmark tool “Altmetric it!” (the tool can be downloaded from https://www.altmetric.com/products/free-tools/bookmarklet/). Online mentions of an article are picked up and collated by an automated algorithm program. The program calculates AS and presents it in the center of a colored donut figure. Each color of the donut represents a distinct online platform (LinkedIn, Twitter, mainstream media, blogs, etc.) (Figure 1).

Statistical analysis

The Kolmogorov-Smirnov test was performed for the evaluation of the distribution of numerical data. As the data were not normally distributed, the median and 25-75% interquartile range were used. Spearman’s correlation analysis test was performed for AS, citation number, ACpY, h-index, impact factor, five-year impact factor, immediacy index, Eigenfactor score, normalized Eigenfactor score, article influence score, journal citation indicator, published year, and the number of years since publication. Categorical data were expressed as percentages or numbers. Statistical analysis was performed with Statistical Package for Social Sciences (SPSS) software (version 26; IBM Corp., Armonk, NY). A value of p < 0.05 was accepted as statistically significant.

## Results

The WoS database scan revealed 12,198 articles on VRS between 2010 and 2020. The 100 most cited (T100) articles had citation numbers ranging from 55 to 368, with a median value of 71.5 (Q1: 61 and Q3: 87.75) (Table 1). The most cited article was "Inverted internal limiting membrane flap technique for large macular holes," written by Michalewska in 2010 and published in the “Ophthalmology Journal.” The AS median value of the articles in the T100 list was 0 (Q1: 0 and Q3: 3) (Table 1). The article with the highest AS value was "First-in-human study of the safety and viability of intraocular robotic surgery," written by Edwards TL and published in the journal Nature Biomedical Engineering, which had an AS value of 125.

**Table 1:** Altmetric scores and citation numbers of the top 100 articles, ranked according to the study types Median values (Q1 and Q3, respectively) were used. WoS: Web of Science.

Study type	Number of articles	Altmetrics scores	Times cited (WoS Core)
All article	100	0 (0-3)	71.5 (61-87.75)
Original scientific paper	85	0 (0-3)	73 (60-87.5)
Prospective, randomized clinical trial	5	3 (1-6.5)	81 (65-262.5)
Prospective, interventional study	13	1 (0-5)	58 (56-94)
Prospective, observational study	12	3 (3-5.5)	71.5 (62.25-107.5)
Prospective cohort study	4	0 (0-0)	99 (73.25-133)
Retrospective, interventional study	18	0 (0-4)	67 (56.75-84)
Retrospective, observational study	19	0 (0-1)	74 (64-80)
Retrospective cohort study	1	0	78
Multicenter retrospective study	8	0.5 (0-1.75)	64 (56-75)
Cross-sectional study	2	0	72.5
Experimental study	3	0	104
Systematic review and meta-analysis	3	0	62
Meta-analysis	1	1	57
Review	11	0 (0-4)	70 (65-89)

As the first name, the author with the most articles in the T100 list was Ehlers JP with six articles. Looking at the entire list of authors, Ehlers JP (seven articles), Srivastava SK (five articles), and Hiraoka T (five articles) were the authors with the most articles, respectively. Ehlers JP's articles had a citation number median value of 75 (Q1: 64 and Q3: 109) and AS median value of 4 (Q1: 3 and Q3: 10). The citation median value of Srivastava SK's articles was 75 (Q1: 69 and Q3: 103) and AS median value was 4 (Q1: 3 and Q3: 11). Hiraoka T's articles had a median value of 72 (Q1: 63.5 and Q3: 74.5) and AS median value of 1 (Q1: 0 and Q3: 5).

There was no T100-listed publication from 2020. When other years were examined, the year with the highest altmetric values of the articles was 2018 with 62.5 AS, and the lowest year was 2010 with 0 AS (Table 2). When looking at the citation numbers, it was seen that the year with the highest median value was 2016 with 89.5 citation median values and the year with the least citation median value was 2013 with 65 citation median values (Table 2).

**Table 2:** Altmetric scores and citation numbers of the top 100 articles, ranked according to publication years Median values (Q1 and Q3, respectively) were used.

Published years of articles	Number of articles	Altmetrics scores	Citations
2010	22	0 (0-0)	75.5 (63.5-132.5)
2011	20	0.5 (0-3)	68 (58-93.25)
2012	20	0 (0-1)	73 (58-79.5)
2013	14	0.5 (0-3)	65 (61.75-93.25)
2014	12	4 (0-9.25)	74.5 (61.75-97.5)
2015	4	0.5 (0-1.75)	67.5 (59.25-75)
2016	4	6 (1.5-44.25)	89.5 (65.25-101.75)
2017	1	3	56
2018	2	62.5	55
2019	1	1	58
2020	-	-	-

The articles in the T100 list were written by 84 first-name authors and were published in a total of 17 journals. The journal with the most articles on the T100 list was “Retina-The Journal of Retinal and Vitreous Diseases” with 27 articles (Table 3). "Nature Biomedical Engineering" was the journal with the highest impact factor (IF) value, and the journal with the highest h-index value was “Ophthalmology” (Table 4). According to the SCImago Journal & Country Rank, the number of journals in category Q1 was 13, and there were two journals in both categories Q2 and Q3. A total of 91 articles were published in Q1-class journals, seven articles were published in Q2-class, and two articles were published in Q3-class journals (Table 4). Looking at the topics of the articles, most publications were on macular hole, epiretinal membrane, and retinal detachment, respectively (Table 5). The most common type of articles were original scientific papers (85), followed by 11 reviews, three systematic reviews and meta-analyses, and one meta-analysis article.

**Table 3:** Some metrics of the journals with the top 100 articles, ranked according to the number of articles Median values (Q1 and Q3, respectively) were used. ^a ^NYsP: number of years since publication; ^b ^ACpY: average citations per year. WoS: Web of Science.

Journals	Number of article(s)	Times cited (WoS Core)	Times cited (All databases)	NYsP^a^	ACpY (WoS Core^b^)	ACpY (All databases)	Altmetric score
Retina-The Journal of Retinal and Vitreous Diseases	27	70 (60-81)	73 (64-88)	9 (8-10)	6.77 (5.91-7.3)	6.88 (6.27-7.8)	2 (0-6)
Ophthalmology	22	82 (70.25-110.25)	86.5 (74.75-115.5)	10.5 (9-12)	12.12 (10.54-15.93)	13.03 (11.15-16.72)	0 (0-2)
American Journal of Ophthalmology	16	71.5 (57.5-103.5)	78.5 (62.5-106.5)	10 (8.25-10.75)	7.6 (7.02-8.1)	8.25 (7.36-9.02)	0 (0-3.25)
Investigative Ophthalmology & Visual Science	10	75.5 (69-114.75)	79 (72.75-118.75)	11 (9.75-12)	8.85 (7.78-10.09)	9.41 (8.32-10.45)	0 (0-3)
Graefe's Archive for Clinical and Experimental Ophthalmology	5	57 (56-72.5)	60 (59.5-75.5)	11 (9.5-11.5)	6.33 (6.01-13.73)	7.44 (6.18-14.09)	0 (0-0)
British Journal of Ophthalmology	4	61.5 (58-65)	68 (60.25-69)	11 (10.25-11.75)	5.09 (4.77-5.47)	5.4 (5.02-5.75)	1.5 (0.25-2.75)
JAMA Ophthalmology	3	66	70	11	5	5.33	3
Survey of Ophthalmology	2	101.5	110	11	6.52	6.95	1.5
Acta Ophthalmologica	2	65.5	67	10.5	5.7	6.1	0
Biomed Research International	2	62.5	65.5	8	10.31	10.81	3
Optics Letters	1	103	107	12	5	5.08	3
Acta Biomaterialia	1	70	74	11	5.18	5.45	3
Biomacromolecules	1	66	68	7	14	14.75	0
Eye	1	66	68	9	11.2	11.4	1
International Ophthalmology	1	61	64	8	5.5	5.9	0
Japanese Journal of Ophthalmology	1	60	64	12	5	5	0
Nature Biomedical Engineering	1	55	59	4	5	5.18	125

**Table 4:** Additional metrics of the journals with the top 100 articles, ranked according to the number of articles WoS: Web of Science.

Journals	Impact factor	5-year impact factor	Immediacy index	Eigenfactor score	Normalized Eigenfactor	Article influence score	Journal citation indicator	% of citable open access	H-index	Q category of WoS
Retina-The Journal of Retinal and Vitreous Diseases	4.256	4.742	1.822	0.02053	4.30481	1.297	1.64	6.41	120	Q1
Ophthalmology	12.079	11.015	5.798	0.04183	8.76962	3.478	4.01	26.96	244	Q1
American Journal of Ophthalmology	5.258	5.729	1.582	0.02621	5.49503	1.824	2.04	13.10	186	Q1
Investigative Ophthalmology & Visual Science	4.799	4.847	0.739	0.05007	10.49620	1.323	1.59	99.58	218	Q1
Graefe's Archive for Clinical and Experimental Ophthalmology	3.117	2.97	1.169	0.01108	2.32444	0.771	1.13	14.86	101	Q2
British Journal of Ophthalmology	4.638	4.554	2.832	0.02125	4.45443	1.347	1.72	24.69	153	Q1
JAMA Ophthalmology	7.389	7.977	3.942	0.02143	4.49325	2.781	2.64	10.60	196	Q1
Survey of Ophthalmology	6.048	5.703	2.431	0.00485	1.01855	1.595	1.07	11.24	132	Q1
Acta Ophthalmologica	3.761	4.18	1.281	0.01340	2.80900	1.140	1.27	20.13	87	Q1
Biomed Research International	3.411	3.62	0.431	0.08141	17.06483	0.745	0.61	99.54	126	Q2
Optics Letters	3.776	3.634	0.900	0.06927	14.52101	0.921	1.34	15.72	272	Q1
Acta Biomaterialia	8.947	9.000	2.326	0.04626	9.69746	1.535	1.76	7.20	190	Q1
Biomacromolecules	6.988	6.813	1.701	0.02597	5.44400	1.179	1.60	11.77	220	Q1
Eye	3.775	3.889	1.119	0.01291	2.70802	1.135	1.18	20.13	98	Q1
International Ophthalmology	2.031	1.896	0.442	0.00514	1.07747	0.494	0.67	5.88	45	Q3
Japanese Journal of Ophthalmology	2.447	2.291	0.602	0.00195	0.40947	0.575	0.85	0	56	Q3
Nature Biomedical Engineering	25.671	26.355	6.935	0.0200	4.20596	8.986	5.42	0	56	Q1

**Table 5:** Correlations between metrics * P-value < 0.05; the upper values represent the r-value and the lower values represent the p-value. ACpY: average citations per year; NYsP: number of years since publication; WoS: Web of Science.

	Times cited (WoS Core)	Times cited (All databases)	Publication year	NYsP	Impact factor	5-year impact factor	Immediacy index	Eigenfactor score	Normalized Eigenfactor	Article influence score	Journal citation indicator	% of citable open access	H-index	ACpY (WoS Core)	ACpY (All databases)	Altmetric score
Times cited (WoS Core)	1															
Times cited (All databases)	0.985 <0.001*	1														
Publication year	-0.152 0.131	-0.167, 0.096	1													
NYsP	0.152 0.131	0.167, 0.096	-1	1												
Impact factor	0.285 0.004*	0.281, 0.005*	-0.104, 0.302	0.104, 0.302	1											
5-year impact factor	0.293 0.003*	0.290, 0.003*	-0.054, 0.591	0.054, 0.591	0.975, <0.001*	1										
Immediacy index	0.138 0.171	0.138, 0.170	-0.013, 0.897	0.013, 0.897	0.644, <0.001*	0.604, <0.001*	1									
Eigenfactor score	0.297 0.003*	0.271, 0.006*	-0.165, 0.102	0.165, 0.102	0.583, <0.001*	0.581, <0.001*	0.050, 0.621	1								
Normalized Eigenfactor	0.297 0.003*	0.271, 0.006*	-0.165, 0.102	0.165, 0.102	0.583, <0.001*	0.581, <0.001*	0.050, 0.621	1	1							
Article influence score	0.260 0.009*	0.263, 0.008*	-0.124, 0.219	0.124, 0.219	0.967, <0.001*	0.937, <0.001*	0.657, <0.001*	0.554, <0.001*	0.554, <0.001*	1						
Journal citation indicator	0.376 <0.001*	0.167, 0.097	-0.008, 0.938	0.008, 0.938	0.842, <0.001*	0.845, <0.001*	0.773, <0.001*	0.377, <0.001*	0.377, <0.001*	0.899, <0.001*	1					
% of citable open access	0.226 0.023*	0.188, 0.061	-0.276, 0.005*	0.276, 0.005*	0.441, <0.001*	0.381, <0.001*	-0.028, 0.784	0.788, <0.001*	0.788, <0.001*	0.432, <0.001*	0.167, 0.097	1				
H-index	0.376 <0.001*	0.351, <0.001*	-0.221, 0.027*	0.221, 0.027*	0.829, <0.001*	0.804, <0.001*	0.397, <0.001*	0.867, <0.001*	0.867, <0.001*	0.786, <0.001*	0.625, <0.001*	0.714, <0.001*	1			
ACpY (WoS Core)	0.336 0.001*	0.309, 0.002*	-0.058, 0.564	0.058, 0.564	0.506, <0.001*	0.549, <0.001*	0.206, 0.040*	0.524, <0.001*	0.524, <0.001*	0.457, <0.001*	0.369, <0.001*	0.568, <0.001*	0.596, <0.001*	1		
ACpY (All databases)	0.336 0.001*	0.308, 0.002*	-0.057, 0.576	0.057, 0.576	0.508, <0.001*	0.550, <0.001*	0.208, 0.038*	0.518, <0.001*	0.518, <0.001*	0.460, <0.001*	0.371, <0.001*	0.562, <0.001*	0.594, <0.001*	0.993, <0.001*	1	
Altmetric score	0.091 0.367	0.109, 0.278	0.328, 0.001*	-0.328, 0.001*	-0.054, 0.590	-0.050, 0.619	0.098, 0.330	-0.089, 0.380	-0.089, 0.380	-0.039, 0.698	0.031, 0.756	-0.269, 0.007*	-0.129, 0.202	-0.220, 0.028*	-0.236, 0.018*	1

Correlations between AS, citation numbers, publication year, number of years since publication (NYsP), impact factor, five-year impact factor, immediacy index, Eigenfactor score, normalized Eigenfactor score, article influence score, journal citation indicator, h-index, and ACpY values are presented in Table 6. It was determined that AS was weakly positively correlated with publication year, and weakly negatively correlated with NYsP, percent of citable open access, and ACpY values (Table 6).

**Table 6:** Altmetric scores and number of citations of the top 100 articles, ranked according to the study topics Median values (Q1 and Q3, respectively) were used. ERM: epiretinal membrane; ILM: internal limiting membrane; DRP: diabetic retinopathy; OCT: optical coherence tomography.

Study topics	Number of articles	Altmetrics scores	Citations
Macular hole	22	0 (0-3.25)	82.5(66-135.5)
ERM	16	0 (0-0)	66.5 (57.75-89.5)
Vitreomacular traction	4	0 (0-7.5)	81 (65.25-129)
Macular hole + ERM	2	4	80.5
Vitreomacular traction + macular hole	1	3	81
ILM peeling	2	2	65.5
Submacular hemorrhage	3	1	57
Retinal detachment	14	0 (0-1.25)	72.5 (61.5-77.25)
DRP	5	0 (0-2)	61 (59.72.5)
Vitreous substitutes	6	1.5 (0-3.75)	68 (63.75-86)
Small-gauge pars plana vitrectomy	4	0 (0-0.75)	88 (66-139.25)
Intraoperative OCT	6	3.5 (3-7.5)	106 (88.5-110.75)
Optic pit	3	7	57
Others	12	0.5 (0-12.75)	60 (56-66)

## Discussion

In this study, we found that there was no significant relationship between the AS and the number of citations in the detailed metric analysis of the 100 most cited articles on VRS published in the last 10 years. It was noteworthy that there was a significant negative correlation between the AS and the time since the publication of the article. This shows that more recent articles are circulated more in the online world.

Social media is a comprehensive and dynamic communication tool, and it can mediate the communication and collaboration of colleagues and scientific communities from different regions [13,14]. It can assist in the rapid dissemination of scientific knowledge, as well as enable discussion of a publication online and the emergence of new ideas [14,15]. Online communication tools are increasingly used by scientific journals, academic institutions, and scientists. Many journals now have social media accounts where they share articles with their followers. In this way, they aim to render it possible for an article to reach a wider audience, to be downloaded and viewed more [4,5]. It has been shown in a study that the social media accounts of the journals in the Q1 category in the SCImago journal ranking (SJR) are followed by more users compared to the journals in the lower categories [4]. When calculating the AS, follower numbers of social media accounts are not taken into account. However, reaching more followers can trigger more downloads, sharing, and comments on the article.

Altmetrics, a web-based metrics system, can provide insight into how much attention an article receives online. Recently, many journals offer the AS of their articles in addition to traditional metrics on their web pages [16]. According to Haustein et al., topics of public interest may differ from those of scientists, and an over-cited article may not receive enough public attention [17]. Although an article receives great attention on social media, the low number of citations it receives does not indicate that altmetric analysis is worthless [18]. On the contrary, this situation provides a new perspective in measuring the value of the study. It shows that the study has reached the relevant audience and attracted attention even if the article is not yet sufficiently noticed by the academic community [18,19]. According to Garcia-Villar, the AS can be defined as the "social impact factor" of an article [1]. It has been shown that issues concerning public health and public interest attract more attention and receive more interaction, while more technical and specific topics do not receive sufficient responses on social media [9]. In our study, it was determined that almost half of the articles included in the T100 list were related to surgery for vitreoretinal interface diseases. However, it was observed that the topic with the highest citation numbers and highest AS was related to intraoperative optical coherence tomography. We believe that the low AS of the articles included in our study is due to the fact that the subject is a specific issue that concerns health professionals.

The journal with the highest number of articles on the T100 list was "Retina-The Journal of Retinal and Vitreous Diseases." We think that this is because the “Retina Journal” is dedicated specifically to vitreoretinal diseases, whereas virtually all other journals were comprehensive ophthalmology journals covering also ophthalmology topics other than vitreoretinal diseases/surgery.

In a recent study examining the ophthalmology literature, it was determined that there is no close relationship between traditional metrics methods and alternative metrics [20]. In another study on glaucoma, the AS values of the 50 most cited articles were found to range from 176 to 0, and there was a significant positive correlation between citation numbers and AS [21]. Conversely, in a study on retinal diseases, Sener et al. found that the AS values of the 100 most cited articles were ranging from 1340 to 0, but no correlation was found between the number of citations and AS [9]. Similar to this study, in our study, we observed that there was no correlation between citation count and AS. However, the articles in this study were mainly on the medical retina and included only five articles on VRS. Thus, we think that this study represents only a small minority of the popular VRS articles. In another study evaluating 100 articles on glaucoma with the highest AS values, no correlation was found between the journal IF and AS [7]. Likewise, there was no correlation between IF and AS in our study.

Altmetric analysis is not just a simple sum of mentions. In addition to the mention counts, the source and the authors of the mentions are of great importance. Different sources have different weights in the score calculation. In the altmetric attention score report, the sources of the mentions contributing to the total score are presented in detail. This allows not only quantitative but also qualitative evaluation of the article. For instance, news about research in the mainstream media gets a higher score than a mention on Twitter or Facebook since it is likely to be seen by more people [20]. The author of the mentions is evaluated in the score calculation to avoid bias in favor of a particular article or journal. Self-mentions of a journal or author have less impact on the score than a mention of an influential researcher who is not an author of the article. Repeated mentions of a study by the same account are ignored in the score calculation [8,22,23].

There are some limitations of our study. First, our study included not all articles on VRS, but the 100 most cited articles on this subject. Therefore, all studies on this subject may not be represented. Another limitation is that since the use of social media has been more intense in the last 10 years, only the articles published in this period were included in our study. Even if they made a significant impact in their field, previously published articles may not have been included in our study. Studies with high AS values published in recent years may not have entered our T100 list because they had not yet reached enough citation numbers. Therefore, we cannot provide information on which of the recently published articles have the highest AS scores. However, in this study, we determined the articles in terms of the number of citations, which is still the most accepted metric measurement by academic scholars.

## Conclusions

In conclusion, to our knowledge, this is the first study on altmetric analysis of the most cited articles on VRS. In our opinion, altmetric analysis is not a competitor to traditional metric methods, but complementary to them. None of the metrics systems are flawless. Traditional metrics methods are based on citation counts. However, a considerable period is required for the accumulation of citations. In turn, altmetric analysis allows authors and journals to track the social interactions and online circulation of an article from the moment it is published. Altmetric analysis can help identify more intriguing research topics and can prompt more academic studies on these topics. Nevertheless, an article with a high AS value may not always be of good quality. For instance, an article having a bad reputation and negative mentions on online networking platforms may have a high AS value. Likewise, based on the findings in our study, it is clear that a low AS does not indicate that an article is of low scientific value. The AS alone is not a reliable indicator of the scientific value of an article and should be evaluated in conjunction with traditional metrics. Altmetric analysis has aspects that need improvement. However, as online communication platforms become more popular for article sharing, the use of alternative metric methods seems inevitable.
